# Supplementary material for: The genome sequence of the Figwort Mason Wasp, Symmorphus gracilis (Brulle, 1832) (Hymenoptera: Vespidae)
Source: Wellcome Open Res. 2026 Mar 31;11:199. [Version 1] doi: 10.12688/wellcomeopenres.26161.1 (PMC13153769; doi:10.12688/wellcomeopenres.26161.1)
Supplement: Peer review history

# Reviewer report: Reviewer response for version 1

**DOI:** 10.21956/wellcomeopenres.28810.r153842

**Published:** 2026-05-07

**Authors:** Ralph S Peters

**Affiliations:** 1 Leibniz Institute, Bonn, Germany

The manuscript accompanies publication of a chromosome-level genome of a representative of the solitary Vespidae subfamily Eumeninae (potter wasps, mason wasps). The manuscript and genome publication merely provides a resource for future research and provides no own studies on taxonomy, evolutionary biology or others. This, however, is no problem, as genome resource papers are common, and a high quality genome (even including two haplotypes from the same individual female) from a broad representation of the megadiverse Hymenoptera is highly welcome, including the mason wasps Symmorphus.

 The background section with some info and intro to mason wasps and the genus Symmorphus provides information maybe even in too much detail (for example, in the description of differences between S. gracilis with congeners that is not really necessary here and also not ideally structured). Also, the end of the background section indicating some possible applications of the provided genome is quite generic (though not wrong) and could use some more specific ideas, for example, for studies within Vespidae. Ergo, the authors should consider shortening the section on diagnosis of S. gracilis (and rather refer to the literature) and extending the section on possible applications of the provided genome.

 The methods and results (there is no discussion which is fine for a genome resource paper) are sufficient and reflect the current state-of-the-art of producing high-quality genomes.

Are sufficient details of methods and materials provided to allow replication by others?

Yes

Is the rationale for creating the dataset(s) clearly described?

Yes

Are the datasets clearly presented in a useable and accessible format?

Yes

Are the protocols appropriate and is the work technically sound?

Yes

Reviewer Expertise:

Hymenoptera diversity and evolution. phylogenomics, sometimes genomics

I confirm that I have read this submission and believe that I have an appropriate level of expertise to confirm that it is of an acceptable scientific standard.

# Reviewer report: Reviewer response for version 1

**DOI:** 10.21956/wellcomeopenres.28810.r153205

**Published:** 2026-04-30

**Authors:** Sharu Paul Sharma

**Affiliations:** 1 Iowa State University, Iowa, USA

The manuscript presents Figwort Mason Wasp ( *S. gracilis*) genome assembly as a part of the Darwin Tree of Life project. The study provides a potentially useful genomic resource for future work in this and related taxa.

 Overall, the manuscript is clear and concise with metrics expected of a high quality assembly. There are a few points that need clarification before publication.

 - The authors should briefly discuss why the final assembly span is substantially larger than the GenomeScope2 haploid genome size estimate.

 - The assembly meets most high-level quality criteria, but the contig N50 falls below the benchmark reported in Table 4. The authors should briefly acknowledge this and clarify whether it affects downstream use of the assembly.

 - The authors should clarify how users should interpret the lower k-mer completeness of each individual haplotype relative to the benchmark, especially since the combined haplotypes achieve high completeness. This is important for users who may rely primarily on haplotype 1.

Are sufficient details of methods and materials provided to allow replication by others?

Yes

Is the rationale for creating the dataset(s) clearly described?

Yes

Are the datasets clearly presented in a useable and accessible format?

Yes

Are the protocols appropriate and is the work technically sound?

Yes

Reviewer Expertise:

Bioinformatics

I confirm that I have read this submission and believe that I have an appropriate level of expertise to confirm that it is of an acceptable scientific standard.
